# Diffusely adherent *Escherichia coli* strains isolated from children and adults constitute two different populations

**DOI:** 10.1186/1471-2180-13-22

**Published:** 2013-02-01

**Authors:** Rosane Mansan-Almeida, Alex Leite Pereira, Loreny Gimenes Giugliano

**Affiliations:** 1 Laboratório de Microbiologia, Departamento de Biologia Celular, Universidade de Brasília, Brasília, DF, 70910-900, Brazil

**Keywords:** Diffusely adherent *Escherichia coli*, Diarrhea, Microbiota

## Abstract

**Background:**

Diffusely adherent *Escherichia coli* (DAEC) have been considered a diarrheagenic category of *E. coli* for which several potential virulence factors have been described in the last few years. Despite this, epidemiological studies involving DAEC have shown inconsistent results. In this work, two different collections of DAEC possessing Afa/Dr genes, from children and adults, were studied regarding characteristics potentially associated to virulence.

**Results:**

DAEC strains were recovered in similar frequencies from diarrheic and asymptomatic children, and more frequently from adults with diarrhea (P < 0.01) than from asymptomatic adults. Association with diarrhea (P < 0.05) was found for SAT-positive strains recovered from children and for curli-positive strains recovered from adults. Mixed biofilms involving DAEC and a *Citrobacter freundii* strain have shown an improved ability to form biofilms in relation to the monocultures. Control strains have shown a greater diversity of Afa/Dr adhesins and higher frequencies of cellulose, TTSS, biofilm formation and induction of IL-8 secretion than strains from cases of diarrhea in children.

**Conclusions:**

DAEC strains possessing Afa/Dr genes isolated from children and adults represent two different bacterial populations. DAEC strains carrying genes associated to virulence can be found as part of the normal microbiota present in asymptomatic children.

## Background

*Escherichia coli* is one of the most frequent causes of diarrhea in children in developing countries. However, characterization of truly diarrheagenic groups or strains can be a complex task because this species is one of the first colonizers of the human gut. Moreover, wild strains exhibit great genetic plasticity and heterogeneity [1].

Diffusely adherent *Escherichia coli* have been considered a diarrheagenic group of *E. coli* (DEC). They are characterized by the diffuse adherence pattern on cultured epithelial cells HeLa or HEp-2 [2]. Approximately 75% of DAEC harbor adhesins from the Afa/Dr family, responsible for this adherence phenotype [3]. Since Germani *et al.*[4] demonstrated that, among DAEC strains, only those that were positive to *daaC* probe - that recognize a conserved region from Afa/Dr adhesins operons - were found in higher frequency in diarrheic patients than asymptomatic controls, much attention has been given to DAEC strains possessing Afa/Dr adhesins.

The adhesins of Afa/Dr family have been implicated in DAEC pathogenesis. They include adhesins found in uropathogenic strains, like the Dr adhesin, in addition to AfaE-I, AfaE-II, AfaE-III, AfaE-V and F1845, which occur in diarrheagenic DAEC strains [5]. They recognize DAF (Decay Accelerating factor, CD55) and some of them also recognize CEACAMs (CEA-related molecules) as receptors [3]. The receptor is recruited around the bacteria after binding to the host cell [6,7]. The binding of strains expressing F1845 or Dr adhesin can promote the dismantling of the actin network in intestinal cells, causing elongation of microvilli [8,9] and redistribution of cytoskeleton-associated proteins in HeLa cells [10].

However, in some studies, DAEC Afa/Dr^+^ strains are isolated from cases of diarrhea and controls in similar frequencies [11,12], suggesting that additional factors may be necessary to trigger disease. In recent years, some of these potential virulence factors have been described. In addition, some studies have implicated DAEC strains as diarrheal agents only in children older than six months, depending on the study, and in adults. [4,13-19].

Evidence of a type three secretion system (TTSS) in DAEC Afa/Dr^+^ isolated from cases of diarrhea in children has been demonstrated by Kyaw *et al.*[20]. The concomitant presence of Afa/Dr adhesins in these strains suggests that an adhesin-receptor-effector protein mechanism, similar to the one seen in EPEC (enteropathogenic *E. coli*), might occur in DAEC. After adhesion and intimate contact, EPEC strains use TTSS to inject effector proteins into the host cell, inducing lesions in the cytoskeleton.

Taddei *et al.*[21] reported the presence of the secreted autotransporter toxin (SAT) belonging to the family of serine protease autotransporters of *Enterobacteriaceae* (SPATE) in DAEC strains isolated from diarrhea. Guignot *et al.*[22] have demonstrated that SAT is able to cause lesions on tight junctions of epithelial cells, which in turn may lead to an increase in their permeability. They also found SAT more frequently in DAEC strains isolated from diarrheic children than from asymptomatic subjects, corroborating the role of SAT as a virulence factor.

DAEC strains have demonstrated pro-inflammatory effects, related to an increased secretion of interleukin-8 by epithelial cells. In T84 cells infected by wild-type strains, basolateral secretion of IL-8 promotes transmigration of polymorphonuclear leukocytes (PMNLs) across the epithelial monolayer [23]. The transmigrated PMNs increase apoptotic rates and reduce phagocytic activity [24] which can contribute to maintain the inflammatory response without eliminating the pathogen. Some studies [18,25] have found that the ability of increasing IL-8 secretion in epithelial cells by DAEC strains was associated with diarrhea in children.

One characteristic that has not been studied in DAEC is the ability to form biofilms. Although biofilm formation is a widespread phenomenon in bacteria, only recently has the importance of biofilms as a pathogenic factor been demonstrated for *E. coli,* such as in atypical EPEC strains [26] and in enteroaggregative *E. coli* (EAEC). The latter have biofilm formation as the only consensual virulence factor [27].

In a previous study performed in this laboratory [28], it was found that EAEC biofilms could be enhanced by interaction with a *Citrobacter freundii* strain isolated concomitantly with EAEC from a diarrheic child. These mixed biofilms seem to be mediated by F pili. Aside from their role in conjugation, F pili have been considered important in establishing *E. coli* biofilms, in addition to other components like curli and cellulose [29,30]. In this work, we employed a *C. freundii* strain to assess the capacity of DAEC strains to form biofilms alone or with other bacteria, as well as to investigate the occurrence of synergistic effects as seen for EAEC strains. Furthermore, we analyzed characteristics potentially associated with virulence or related to biofilm formation, such as Afa/Dr adhesins, SAT toxin, TTSS, F pili, curli, cellulose and stimulus of IL-8 secretion by epithelial cells. The aim of this work is to study the overall profile of DAEC strains isolated from children and adults, both from cases of diarrhea and controls, thereby performing a systematic study of DAEC.

## Results

### Prevalence of DAEC strains

A total of 1,253 *E. coli* isolates recovered from stool samples of 127 cases of diarrhea in children and 127 asymptomatic controls were examined for the presence of genes belonging to the conserved region of the *afa operons* (*afa*B-C), which encode the Afa/Dr family of adhesins. Since EPEC strains occasionally have these adhesins, the presence of *eae* gene, typical of this category, was also investigated. One hundred and eighteen *afa*B-C positive isolates tested negative for *eae.* In adhesion tests, most strains (95/118 - 80.5%) showed diffuse adherence. Nine strains (7.7%) were non-adherent and one strain (0.8%) adhered in an unclassified pattern. These strains were excluded from the study. Despite the fact that other thirteen strains (11%) caused cell detachment, diffusely adhering bacteria could be detected in remaining cells, and these strains were included in the sample. Thus, one hundred and eight strains, including 50 from cases of diarrhea and 58 from controls, were considered as DAEC possessing Afa/Dr genes (Table 1).

**Table 1:** DAEC strains possessing Afa/Dr genes detected among patients and controls

**Group**	**Children**		**Adults**		**Total**
	**Diarrhea**	**Control**	**Diarrhea**	**Control**	
Number of subjects enrolled	127	127	143	119	516
Number of subjects harboring DAEC	21 (16.5%)	25 (19.6%)	27* (18.9%)	5* (4.2%)	78 (15.7%)
Number of DAEC strains isolated in each group	50	58	27	15	150

*(P < 0.01).

The prevalence of DAEC possessing Afa/Dr genes in cases of diarrhea in children and their controls was similar (Table 1). DAEC strains were detected in 21 of the 127 cases of diarrhea in children (16.5%), and in 25 of 127 asymptomatic controls (19.6%). Association with diarrhea was not found even when the children were stratified by age (comparing children younger or older than six months, as well as 12 months).

Furthermore, DAEC strains possessing Afa/Dr genes were recovered from 27 out of 143 (18.8%) cases of diarrhea in adults, and from five out of 119 (4.2%) healthy subjects (Table 1). All strains showed diffuse adherence in adhesion tests. Consequently, DAEC strains were found in higher prevalence in cases of diarrhea in adults (P < 0.01). Twenty seven DAEC strains were obtained from adults with diarrhea and fifteen from asymptomatic adults (Table 1).

### Diversity of Afa/Dr adhesins in strains from children and adults

Some molecular studies indicate the involvement of Afa/Dr adhesins in DAEC-induced pathogenesis [8-10]. We examined their distribution in strains isolated from cases of diarrhea and asymptomatic controls. Different members of the Afa/Dr family were detected using specific primers for the adhesin-encoded genes (*afa*E). Their distribution is shown in Table 2.

**Table 2:** Afa/Dr adhesins distribution in DAEC strains isolated from cases of diarrhea and asymptomatic controls

	**Strains isolated from**					
	**Children**			**Adults**		
	**Diarrhea**	**Control**		**Diarrhea**	**Control**	
***afaE***	**N (%)**	**N (%)**	**Total**	**N (%)**	**N (%)**	**Total**
**1**	22 (44)	19 (32.8)	41 (38)	12 (44.4)	5 (33.3)	17 (40.5)
**2**	5 (10)	3 (5.2)	8 (7.4)	1 (3.7)	2 (13.3)	3(7.1)
**3**	1 (2)	1 (1.7)	2 (1.8)	2 (7.4)	1 (6.7)	3 (7.1)
**5**	1 (2)	2 (3.5)	3 (2.8)	1 (3.7)^a^	7 (46.7)^a^	8 (19)
**X**	12 (24)	7 (12)	19 (17.6)	11 (40.7)^a^	0^a^	11 (40.7)
***daaE***	3 (6)	9 (15.5)	12 (11)	0	0	0
**1 + 2**	5 (10)	0	5 (4.6)	0	0	0
**1 + 3**	0	1 (1.7)	1 (0.9)	0	0	0
**1 + 5**	0	6 (10.3)	6 (5.1)	0	0	0
**1 +** ***daaE***	1 (2)	5 (8.6)	6 (5.1)	0	0	0
**1 + 2 +** ***daaE***	0	1 (1.7)	1 (0.9)	0	0	0
**2 +** ***daaE***	0	2 (3.5)	2 (1.8)	0	0	0
**5 +** ***daaE***	0	2 (3.5)	2 (1.8)	0	0	0
**Total**	**50**	**58**	**108**	**27**	**15**	**42**

^a^P < 0.05 (cases x control).

In 20% (30/150) of *afa*B-C-positive strains, the adhesin gene could not be identified. These strains with indeterminate *afa*E were referred to as “*afa*-X”.

Strains isolated from children and adults exhibited a very different distribution of Afa/Dr adhesin encoding genes. The *afaE*-1 gene was a notable exception, being similarly distributed for all groups. It was also the most frequent gene.

Strains isolated from children showed great diversity of adhesins. More than one type of Afa/Dr adhesins were detected in 21.3% (23/108) of strains isolated from children, and in 29.3% (17/58) of strains isolated from asymptomatic children. All genetic combinations involve *afaE*-1 or *daaE*. The *afaE*-1/*afaE*-2 association was found only in diarrheagenic strains (P < 0.05). The F1845 encoding gene was only found in strains isolated from children, especially in control strains.

Strains isolated from adults showed a low variability of *afa*E genes. Prevalence of *afa*-X was higher (P < 0.01) in cases of diarrhea, while prevalence of *afa*E-5 was higher in controls (P < 0.01). Neither the *daaE* gene nor associations between two types of adhesins were detected in strains from adults.

### Distribution of virulence factors

DAEC strains were examined regarding characteristics associated with virulence. The percentage of strains carrying virulence genes or possessing phenotypic characteristics associated to biofilm formation is summarized in Table 3.

**Table 3:** Characteristics associated to virulence in DAEC strains possessing Afa/Dr genes isolated from children and adults

	**Strains isolated from N(%)**			
	**Children**		**Adults**	
**Characteristic**	**Diarrhea**	**Control**	**Diarrhea**	**Control**
***traA***	45 (90)	47 (81)	19 (70.3)	13 (86.6)
**Cellulose**	5 (10)	17 (29.3)	1 (3.7)	0
**Curli**	31 (62)	39 (67.2)	16 (59.2)^a^	1 (6.7)^a^
***sat***	23 (46)	11 (18.9)	18 (66.7)	13 (86.6)
**TTSS**	3 (6)	30 (51.7)	1 (3.7)	0
**Motility**	45 (90)	55 (94.8)	19 (70.3)	14 (93.3)
**IL-8 secretion**	14 (28)	31 (53.4)	0^a^	4 (26.6)^a^
**Total**	50	58	27	15

^a^P < 0.05 (cases x control).

As we were interested in investigating a possible role for F pili in the establishment of DAEC biofilms, we performed PCR assays to detect the *traA* gene encoding pilin F. *traA*-positive DAEC strains were frequently detected in all groups of tested strains.

The production of cellulose and curli – common components of *E. coli* biofilms – was investigated. Only one strain isolated from adults tested positive for cellulose production. In strains from children, the prevalence of cellulose production was higher (P < 0.05) among control strains (29.3% - 17/58) than in those recovered from diarrhea (10% - 5/50).

Curli-positive strains were isolated in similar frequencies from diarrheic (62% - 31/50) and asymptomatic (67.2% - 39/58) children. In contrast, in strains from adults, expression of curli was higher (P < 0.05) in strains from diarrhea (59.2% - 16/27) than from controls (6.7% - 1/15).

The gene that codes for the SAT toxin was often found in strains from adults, both diarrheic (66.7% -18/27) and asymptomatic (86.6% -13/15). By contrast, in strains from children, the *sat* gene was found in higher prevalence (P < 0.05) in cases of diarrhea (46%- 23/50) than in controls (18.9% -11/58), corroborating the hypothesis of its involvement in diarrhea induced by DAEC in children.

We also investigated the occurrence of the *escV* and *escJ* genes that are part of the type three secretion system in DAEC strains. When analyzing strains isolated from adults, these genes were found in only one strain, isolated from diarrhea (3.7%). Unexpectedly, 51.7% (30/58) of the strains isolated from asymptomatic children were positive for *escV* or *escJ*, while they were found in only 6% (3/50) of strains from children with diarrhea (P < 0.05).

When the motility of DAEC strains on a semi-solid agar medium was investigated, it was found that most strains (88.6%) had swimming ability, regardless of their origin.

When the strains were tested for IL-8 secretion, 53.4% (31/58) of control strains and 28% (14/50) of strains from diarrhea in children were able to stimulate secretion by HeLa cells (P < 0.01). When analyzing adults, 26.6% (4/15) of strains isolated from asymptomatic control subjects stimulated IL-8 production. All IL-8 stimulating strains isolated from adults came from a single case, and probably represent a clone. Positive strains were not detected in strains isolated from diarrheic adults. The average level of IL-8 secretion by DAEC-stimulated HeLa cells was 60 pg/mL, reaching a maximum of 350 pg/mL. Most strains from both children and adults showed low levels of IL-8 secretion. IL-8 secretion was not detected in non infected HeLa cells or in cells infected with *E. coli* C600.

Ability of stimulate IL-8 secretion in HeLa cells was not associated to motility, *afaE* type or other characteristic examined in this work.

### Classical EPEC serogroups

All strains were screened for classical EPEC serogroups. They were detected in 30.5% (33/108) of DAEC strains isolated from children. We observed serogroups O86, O158, O142 and O127. Serogroup O86 was found most frequently, both in diarrhea and control strains (Table 4). Distribution of genotypic and phenotypic characteristics was similar in DAEC strains belonging to both EPEC and non-EPEC serogroups. Serogroups associated to EPEC were not detected in strains isolated from adults.

**Table 4:** Classical EPEC serogroups found in DAEC possessing Afa/Dr genes isolated from children

**Serogroups**	**O86**	**O127**	**O142**	**O158**	**Non-EPEC**	
	**N (%)**					**Total**
**Diarrhea**	13 (26)	0	1 (2)	5 (10)	31 (62)	50
**Control**	7 (12)	2 (3.4)	0	5 (8.6)	44 (75.8)	58
**Total**	20 (18.5)	2 (1.8)	1 (0.9)	10 (9.2)	75 (69.5)	108

### Biofilms

**Figure 1:**
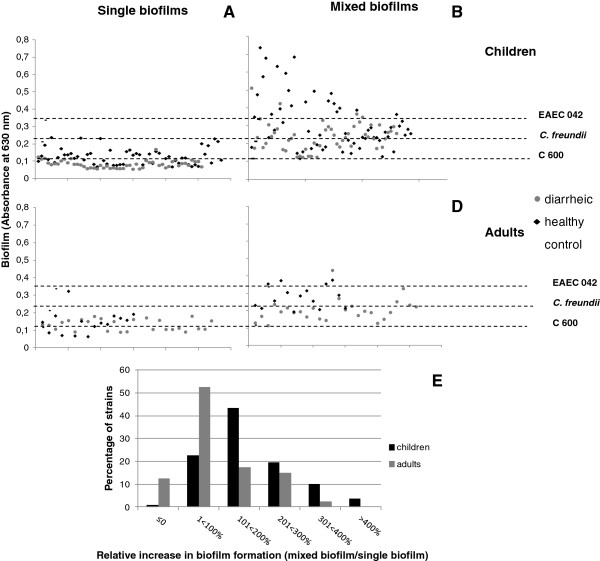
**Effect of interaction DAEC - *C. freundii *in biofilm formation.** Biofilm formation by monocultures of DAEC isolated from children (**A**) and adults (**C**); Increase in biofilm formation in DAEC-*C. freundii* cocultures (**B**, **D**). Comparison between the synergistic effect of cocultivation of DAEC strains recovered from children and adults and an enteroaggregative strain of *Citrobacter freundii* is shown in **E**. The increase in intensity of biofilm formed was higher in consortia involving strains from children.

Most DAEC strains were not able to form biofilms as pure cultures. Tests carried out with DAEC strains isolated from children showed that 88.9% (96/108) of them were unable to form biofilms under the studied conditions; 11% (12/108) formed weak biofilms (Figure 1A). The frequency of strains from children that form biofilms was greater (P < 0.01) in control (18.9% - 11/58) than in cases of diarrhea (2% - 1/50).

Tests performed with DAEC strains isolated from adults showed that 73.8% (31/42) did not form biofilms. Eleven strains (26.2%) formed biofilms (Figure 1C). The frequency of biofilm formation did not differ between cases (25.9% - 7/27) and control (26.6% - 4/15) strains.

The frequency of DAEC strains able to form biofilms was greater (P < 0.05) among strains isolated from adults (26.2% - 11/42) than from children (11% - 12/108).

### Mixed biofilms

In order to evaluate the effect of bacterial combinations on biofilm formation, mixed biofilm assays were conducted using cocultures of DAEC and *C. freundii* strain Cf 205, which forms weak biofilms when in monoculture. Mixed biofilm formation was observed in 83% (90/108) of consortia involving strains from children. In 30% (27/90) of consortia, weak biofilms were formed, while 70% (63/90) of cocultures formed strong biofilms, indicating a synergistic effect of the DAEC- *C. freundii* association (Figure 1B). Strong biofilms were more frequent (P < 0.05) in consortia involving strains from asymptomatic children (67.2% - 39/58) than in those involving cases of diarrhea (48% - 24/50).

Biofilm formation was observed in 80.9% (34/42) of consortia involving strains from adults. Twenty-three consortia (67.6% - 23/34) formed weak biofilms, and 32.3% (11/34) formed strong biofilms (Figure 1D). When analyzing the frequency of overall biofilm formation, we found no statistically significant difference in strains from cases (81.5% - 22/27) and control (80% - 12/15).

To verify the relative increase of intensity in mixed biofilm formation, the optical density (OD) observed in each coculture was compared to the OD obtained by the respective DAEC strain in monoculture. The effect of the DAEC - *C. freundii* association was more pronounced in strains from children. The cocultures involving strains from children showed increases in mixed biofilm formation between 101% and 200%*.* For most strains from adults, the increase was less than 100% (Figure 1E). Furthermore, the maximum increase in intensity observed for adult strains was three-fold while in strains from children it reached six-fold.

### Adhesion to HeLa cells

**Figure 2:**
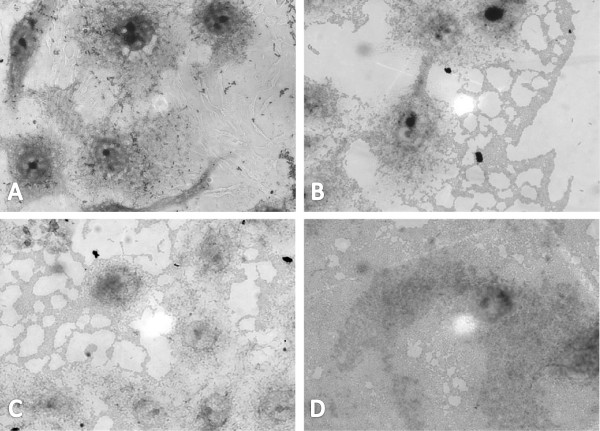
**Adhesion of DAEC and *C. freundii *to HeLa cells.** Adherence to HeLa cell monolayers after 3 hours of incubation is intensified when DAEC and *C. freundii* are inoculated together. **A** – typical diffuse adhesion of DAEC strains, when in monoculture; **B** - enteroaggregative *C. freundii* showing an aggregative adherence pattern, identical to the aggregative adherence of EAEC strains; **C**, **D** - adherence assays with cocultures of *C. freundii* and DAEC.

To evaluate whether the increase in biofilm formation by DAEC – *C. freundii* consortia was associated to an increased adhesion to epithelial cells, mixed adhesion tests were performed. Light microscopy showed that the adhesion to HeLa cells developed by DAEC – Cf 205 associations was greater than that supported by each strain separately (Figure 2). An increment in bacterial adhesion was observed when the experiments were repeated with several DAEC – *C. freundii* pairs that had shown increased biofilms.

### Effect of zinc on mixed biofilms

In order to evaluate the impact of zinc on mixed biofilm formation and, consequently, the role of putative F pili, biofilm assays were performed by adding zinc to the medium. In strains from children, 57.7% (52/90) of DAEC - *C. freundii* consortia had biofilms reduced or abolished when zinc was added. We also observed reduction in 50% of single biofilms (6/12) in the presence of zinc. Similarly, reduction was observed in 52.9% (18/34) of mixed biofilms and 54.5% (6/11) of single biofilms with DAEC strains from adults.

**Figure 3:**
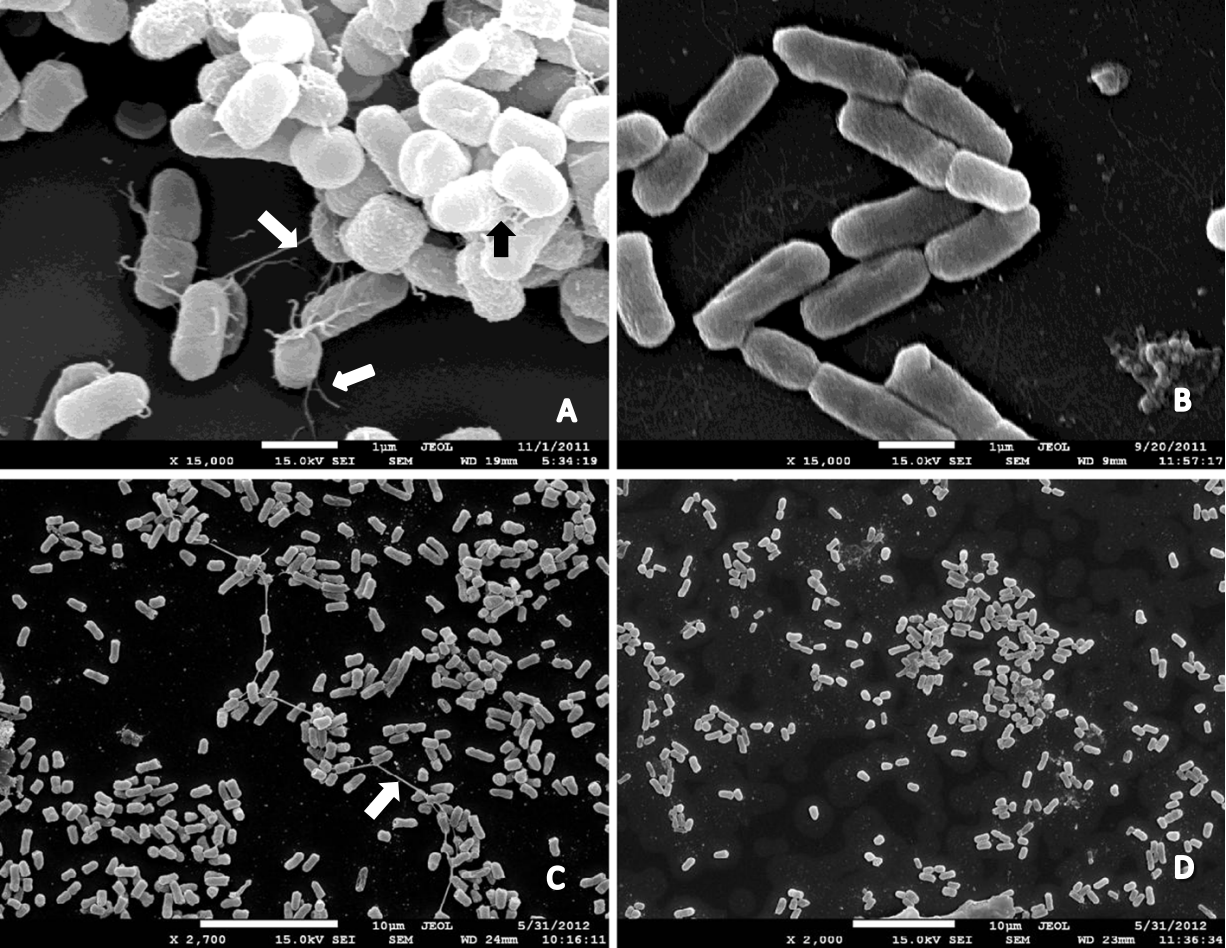
**SEM analysis of mixed biofilms.** Biofilms inhibited by zinc show thick pili (**A**, **C**) that are not visualized in biofilms insensible to zinc (**B**,**D**). Pili are seen mediating cell-to-cell interaction and adhesion to surface (white arrows). Fimbrial structures resembling curli can be observed in some samples (black arrows).

Some mixed biofilms reduced by zinc involving *tra*A positive DAEC strains were submitted to electronic microscopy. The analysis revealed thick, non-bundle forming pili mediating cell-to-cell adherence and adhesion to an abiotic surface (Figure 3A and C). Large amounts of matrix, but not pili, were observed in biofilms that were not affected by 0.25 mM of zinc (3D). Fibers resembling curli were occasionally observed as part of biofilms in addition to pili (3A).

## Discussion

The human gut is colonized by a very complex and diversified microbiota. Bacteria in the gastrointestinal tract play multiple roles in human health, including metabolic features absent in humans [31], modulation of gut morphology and physiology [32] and development of the immune system [33-35]. Colonization begins at birth, but maturation of the microbiota is a continuous process lasting for several years [36-38].

One of the first facultative organisms to colonize the human gut is *E. coli*[39,40]. There is an ongoing debate on whether diffusely adherent *E. coli* (DAEC) represent normal inhabitants of the gut or diarrheagenic strains, because many epidemiological studies have shown inconsistent results [11,14,41]. As the controversy has been attributed, at least in part, to an age factor [13-18] we compared DAEC strains belonging to four different groups: children with diarrhea, asymptomatic children, adults with diarrhea and asymptomatic adults.

We have found remarkable differences between strains isolated from adults and from children regarding the characteristics analyzed in this work.

DAEC strains with undetermined *afaE* were first reported by Zhang *et al.*[42] that described new variants of Afa/Dr adhesins. In 20% (30/150) of *afa*B-C-positive strains in this study, the “E” gene was not identified, and the strains were referred to as “*afa*-X-positive” strains. In the adult group, *afa-X* was only found in strains isolated from cases of diarrhea. This result is similar to that found by Arikawa *et al.*[25], who reported the presence of undetermined *afaE* in 26.3% (5/19) of DAEC strains isolated from cases of diarrhea (which they called “*afaEX”*). In contrast, in another work from Japan [43] the authors found *afaEX* strains isolated from healthy adults. It is unclear if *afa-X* and *afaEX* strains harbor the same or different Afa/Dr adhesins, since the *afaE* gene was not identified. It is likely that there are many yet undescribed variations of Afa/Dr adhesins. Korotkova et al. [44] showed that point mutations in Dr adhesin genes result in phenotypic variability with distinct binding properties. However, in a previous work performed in this laboratory [19] the analysis of surface proteins showed that all *afa-X* strains isolated from diarrheic adults had an identical electrophoretic profile, suggesting that all these strains harbor an identical member of Afa/Dr family. Further studies are required to identify Afa-X and clarify its role in the pathogenesis of diarrheas caused by DAEC in adults.

Strains from adults exhibit few types of adhesins in a characteristic pattern: AfaE-V associated with control and the putative Afa/Dr adhesin Afa-X with diarrhea. In contrast, several types of Afa/Dr adhesins are present in strains from children, with no one in particular associated with disease. Great diversity regarding the types of Afa/Dr adhesins was especially frequent among strains isolated from asymptomatic children, with 29.3% of strains harboring more than one Afa/Dr adhesin. The afaE1 and F1845 adhesins are always present in the associations. Both recognize DAF as a receptor, and F1845 also recognizes CEACAMs [2]. Since adhesins are involved in colonization, the presence of related adhesins able to recognize different receptors could provide an adaptive advantage to these bacteria and explain the apparent redundancy of Afa/Dr adhesins. Interestingly, DAF expression in erythrocytes is higher in adults than in children [45], being especially low in children aged between 24 and 36 months [46]. If this differential expression were also found in enterocytes, it would help explain the advantage of strains from children in presenting adhesins able to bind to receptors other than DAF.

A factor frequently detected in strains isolated from children was the expression of curli. Curli is a bacterial structure involved in the adhesion to both fresh vegetables [47-49] and several proteins widespread in human cells or extracellular matrix, like MHC class I, TLR2, fibronectin and laminin [50]. Most DAEC strains from children that express curli at 37°C were also capable of expressing curli at 28°C (data not shown). Therefore, curli could facilitate further colonization by *E. coli* ingested through food sources mediating attachment once the bacteria are in the body.

By contrast, curli expression was frequent in strains isolated from diarrheic adults but rare in strains from asymptomatic adults, suggesting a potential involvement with diarrheal disease in adults. Several studies have associated curli to virulence of *E. coli*. Besides being a colonization factor [50], curli leads to the stimulation of inflammatory response in its host [50,51], which is mediated by TLR1/TLR2 [52]. Curli was associated to higher rates of invasion of epithelial cells [53] and increased virulence in mice [54].

Curli shares many characteristics with human amyloids [55]. Amyloid deposits induce chronic inflammation, which in turn results in tissue injuries associated with neurodegenerative diseases, with Alzheimer’s disease being the most notorious example. Some lines of evidence suggest that old cells (at least neurons) can be more susceptible to beta-amyloids [56-58]. Analogously, adults could be more susceptible to bacterial amyloids than children, helping to explain why curli might be associated to diarrhea in adults, but not in children. Furthermore, the immune system in children is not fully developed [33], leading us to speculate that while curli expressing *E. coli* strains might be carried by asymptomatic children, healthy adults’ immune systems could exclude those potentially virulent strains.

In EPEC strains, the TTSS is part of the the LEE pathogenicity island [3]. Pathogenicity islands (PAIs) are large portions of microbial genomes that are present in pathogens but not in non pathogenic strains of the same species [59]. In this study, genes that are part of the TTSS apparatus were found in strains isolated from asymptomatic children. Despite considering that they were detected too frequently to be found incidentally, we do not know whether these strains possess a functional TTSS. Blanc-Potard *et al.*[60] found that Afa/Dr DAEC strains C1845 and IH11128 harbor part of a PAI described for an uropathogenic *E. coli* strain. Analogously, some DAEC strains from children could harbor part of a LEE, including part of a TTSS, but not necessarily the complete functional apparatus. Interestingly, TTSS genes were found in strains from children, but not in strains from adults. Many strains from children also belong to some classical EPEC serogroup - again not found in strains from adults - leading us to wonder whether the strains from children may be more closely related to EPEC in evolutionary terms. Although TTSS has been associated to virulence in a broad range of Gram-negative bacteria [61], we have found it in control strains. Even though much emphasis has been given to the role of TTSS in pathogenesis, its presence was recorded in non-pathogenic bacteria such as *Pseudomonas fluorescens*[62] and *Sodalis glossinidius*[63].

By the late fifties, the development of seroagglutination assays enabled the establishment of the classical groups of EPEC. These serogroup-marked strains were frequently associated with sporadic cases of infantile diarrhea as well as outbreaks [64]. In virtue of the current molecular characterization adopted for typing *E. coli* strains, nowadays it is known that some of the so-called classical EPEC serogroups are shared with other diarrheagenic categories [65-67]. The World Health Organization recognized that EPEC comprises strains of 12 O serogroups known as the classical EPEC serogroups: O26, O55, O86, O111, O114, O119, O125,O126, O127, O128, O142 and O158 [68] In this work, we found 30.5% of DAEC isolated from children belonging to serogroups O86, O127, O142 or O158. Serogroup O86 was very frequent, corresponding alone to 20% of DAEC strains isolated from children. This serogroup seems to be widely distributed among different *E. coli* pathotypes, since it has been found in EAEC [65], DAEC [67] and STEC strains [69]. Interestingly, we have not found DAEC strains from adults belonging to EPEC serogroups, reinforcing the differences between DAEC strains isolated from children and adults.

Arikawa *et al.*[25] found that some DAEC strains are able to stimulate IL-8 secretion by epithelial cells and suggested that strains possessing this ability could be implicated in the establishment of diarrhea. The importance of IL-8 stimulus in the pathogenesis of DAEC strains was reinforced by the study of Meraz *et al.*[18]. In a more recent work Arikawa *et al.*[70] found that high levels of IL-8 secretion by epithelial cells were associated with stimulation by flagella via the TLR5 receptor.

We found some DAEC strains stimulating IL-8 secretion by HeLa cells. Meanwhile, association with the motility of strains, and consequently to flagella, was not found, perhaps because almost all DAEC strains in this work were mobile. Interestingly, we found more strains able to stimulate IL-8 secretion cells among strains isolated from asymptomatic children. However, most of DAEC strains stimulated only low levels of IL-8 secretion, which could simultaneously explain the lack of association with diarrhea and the presence of the flagella.

Developing microbiota in children is not formed by random bacterial groups, but instead consisting of bacterial consortia that interact among themselves [71]. Thus, the chance of a given *E. coli* strain establishing itself will be determined, in large part, by the partners previously found in the gut environment and by the relationships among them. A *C. freundii* strain (Cf 205) that was shown to be capable of increasing biofilm formation of EAEC strains isolated from cases of diarrhea was selected from a previous study [28]. Since many DAEC strains were not able to form biofilms alone, or only form weak biofilms, we decided to investigate the effect of Cf 205 in DAEC mixed biofilm assays. Consortia DAEC-*C. freundii* showed not only increased biofilm formation, but also higher adhesion to cultured cells, suggesting that bacterial combinations can be decisive for colonization. A great increase in biofilm formation was observed especially when strains isolated from asymptomatic children were employed in mixed biofilm assays, perhaps because these strains possess greater diversity of adhesins that could help interactions with *C. freundii*. Those strains also showed greater production of cellulose, which is an important component of biofilms, and cellulose could facilitate adherence of bacterial consortia both to abiotic surfaces and cell surfaces.

Other bacterial components possibly involved in formation of mixed biofilms are F pili. It has been demonstrated that the presence of natural conjugative plasmids promotes biofilm formation [29] and that F pili are used in the initial stages of *E. coli* biofilm formation [30]. We believe that F pili are involved in mixed biofilms since most of them were inhibited by zinc in a concentration that does not affect bacterial growth. Furthermore, Pereira *et al.*[28] demonstrated that cell-to-cell interactions involved in EAEC-Cf 205 biofilms were mediated by putative F pili, leading us to hypothesize that F pili also mediate DAEC – Cf 205 biofilms.

The effect of a toxin and the resulting association to diarrhea depend on its effective concentration at the site of infection, which in turn depends on the density of producing bacterial cells. Although SAT is highly prevalent in strains from adults, association with diarrhea was found only in strains from children – precisely the strains which showed greater diversity of adhesins, greater expression of cellulose and biofilm formation, in other words, that possess greatest capacity of colonization.

Recent works in the field of microbial ecology that take advantage of non-cultivating methods are elucidating the gut colonization process. Here, we have found that DAEC strains possessing Afa/Dr genes may reflect some principles that apply to the microbiota in general. First, as microbiota composition is different in children and adults, we found that DAEC from children and from adults represent two different populations, with distinct profiles regarding the characteristics studied in this work. Second, as microbiota seems to be more diversified in control subjects than in diarrhea patients [72], DAEC strains isolated from asymptomatic controls present greater diversity of genes related to virulence. Quiroga *et al.*[73] demonstrated that strains of *E. coli* belonging to four different diarrheagenic categories – including DAEC and EPEC – can be found colonizing infants in the first months of life. Here, we refined the analysis of DAEC strains and found that potentially diarrheagenic strains can be found as part of gut microbiota in children. We also demonstrated that many DAEC strains possessing Afa/Dr genes belong to serogroups associated with EPEC, reflecting perhaps an evolutionary relationship.

DAEC strains as etiological agents of diarrhea are still a matter of controversy. We found that DAEC strains possessing Afa/Dr genes from children and adults possibly possess distinct virulent mechanisms. DAEC strains from children apparently have greater ability of colonizing the gastrointestinal tract, which may contribute to the effective action of a toxin, such as SAT. We also demonstrated for the first time, to the authors’ knowledge, that curli can play a role in pathogenesis of DAEC strains isolated from adults. Further studies are warranted to conclusively demonstrate this involvement.

## Conclusions

DAEC strains possessing Afa/Dr genes isolated from children and adults have shown very distinct profiles regarding the distribution of the characteristics analyzed in this work. Strains from children are more diverse than strains from adults in relation to the studied characteristics. Most characteristics were more frequent in strains from asymptomatic children. In contrast, virulence factors were less frequent in strains from adults, which seem to form a more homogeneous group. Characteristics potentially associated to virulence are distinct in DAEC strains from adults and children. The results confirm the importance of SAT in diarrhea caused by DAEC in children and suggest that its action may be enhanced as a result of their efficiency in colonization. Moreover, curli is a potential virulence factor for DAEC strains that cause diarrhea in adults. Together, these results indicate that DAEC strains possessing Afa/Dr genes isolated from children and adults represent two different bacterial populations. Furthermore, the results also indicate that DAEC strains with virulence markers can be found as part of the normal microbiota present in healthy children.

## Methods

### Specimens and strains

*Escherichia coli* samples belonging to two different collections from this laboratory were used: the first consisted of samples isolated from children under 5 years old treated at two hospitals in Brasília, Distrito Federal, Brazil (*Hospital Universitário de Brasília* and *Hospital Materno-Infantil de Brasília*). One hundred twenty-seven fecal specimens were collected from patients with diarrhea, along with 127 fecal specimens from healthy children. Control subjects were defined as children who did not present diarrhea in the four-week interval preceeding sample collection. The subjects were matched by age and socioeconomic status within 15 days of sample collection. Subjects who had used antibiotics up to 15 days before sample collection were excluded from the study. Diarrhea was characterized by an increased number of evacuations with loose feces. In general, five *E. coli* strains were isolated from each fecal sample, with a total of 1253 isolates recovered.

The second collection used in this study consists of DAEC strains partially characterized in previous studies [19,74] obtained from 143 cases of diarrhea in adults and from 119 control subjects.

For this study we selected *E.coli* samples possessing the conserved region of operons Afa/Dr (afaB/C genes) and negative for *eae* - DAEC . All samples were checked for *Salmonella* and *Shigella* organisms, in which case they were excluded from the study. Any sample where other *E. coli* pathovars - EPEC, EAEC, EIEC, ETEC and EHEC/STEC - were recovered were excluded from this study. The samples isolated from children were also checked for rotavirus.

All *E. coli* strains underwent serological assays for the detection of classical EPEC serogroups O26, O55, O86, O111, O114, O119, O125, O126, O127, O128, O142, O158; EIEC serogroups O28ac, O29, O112ac, O124,O136, O143, O144, O152, O164, O167 and EHEC serogroup O157.

Standard strains of *E. coli* used as control are indicated in Table 5 and were kindly provided by Dr. C. Le Bouguénec and Dr. B. Nowicki. The biofilm-forming aggregative *C. freundii* (EACF) strain 205 used in mixed biofilms was isolated from a child (aged 13 months) on the fifth day of a mucous diarrhea that presented, on average, 15 evacuations per day, during a case–control study [28].

**Table 5:** **Standard *E. coli *strains utilized in this work**

**Strain**	**Characteristic**
C600	Negative control
KS 51	Operons afa/Dr and afaE-1
A 22	afaE-2
A 30	afaE-3
AL 851	afaE-5
IH 11128	Dr hemagglutinin
C 1845	F1845/diffuse adhesion
042	Biofilm/ aggregative adhesion
17.2	Biofilm/ aggregative adhesion

Bacterial strains were preserved at −20°C in a Luria-Bertani broth medium with 15% glycerol.

### Ethics statement

The study was approved by the University of Brasília’s Health Science ethics committee. It was conducted in accordance with guidelines expressed in the Declaration of Helsinki. We sought and obtained individual written informed consent for all subjects; parents or guardians were invited to sign (or thumbprint if illiterate) the informed consent form.

### Detection of virulence markers

Virulence markers were detected by polymerase chain reaction (PCR) performed using the primers listed in Table 6. The cycling conditions for PCR were as follows: 10 cycles at 94°C for 1 min, at 55°C for 1 min, and at 72°C for 90 s, followed by 20 cycles at 94°C for 1 min, at 60°C for 1 min, and at 72°C for 90 s. All target fragments were amplified using similar parameters, except for the annealing temperature. Supernatants derived from bacterial suspension treated by boiling were used as the source of DNA template.

**Table 6:** Primers used in polymerase chain reaction analysis

**Gene**	**Locus description**	**Primer sequence**	**Fragment length**	**Annealing temperature**	**Reference**
***afa*B-C**	Conserved region of Afa/Dr operons	5Â´ CTGGGCAGCAAACTGATAACTCTC 3Â´	750 pb	62°C	[75]
		5Â´ CATCAAGCTGTTTGTTCGTCCGCCG 3Â´			
***afaE*-1**	Afa-I afimbrial adhesin	5Â´ CGAAAACGGCACTGACAAG 3Â´	230 pb	61°C	[19]
		5Â´ AGGCTTCCGTGAATACAACC 3Â´			
***afaE*-2**	Afa-II afimbrial adhesin	5Â´ TTAGACCGTACTGTTGTGTTACC 3	375 pb	48°C	[42]
		5Â´ TTTCCCAGTAGACTGGAATGAAGC 3Â´			
***afaE*-3*/dre***	Afa-III afimbrial adhesin/Dr afimbrial adhesin	5Â´ TTAGACCGTACTGTTGTGTACC 3Â´	408 pb	65°C	[76]
		5Â´ ACCATTGTCGGTCGTCCAGGC 3Â´			
***afaE*-5**	Afa-V afimbrial adhesin	5Â´ TTAGACCGTACTGTTGTGTTACC Â´	429 pb	48°C	[42]
		5Â´ AGCATCGGCGCGGTATACGGT 3Â´			
***daa*E**	F1845 fimbrial adhesin	5Â´ TGACTGTGACCGAAGAGTGC 3Â´	380 pb	48°	[19]
		5Â´ TTAGTTCGTCCAGTAACCCCC 3Â´			
***Sat***	Secreted auto transported toxin	5Â´ GCAGCAAATATTGATATATCA 3Â´	630 pb	57°C	[21]
		5Â´ GTTGTTGACCTCAGCCAAGGAA 3Â´			
***escJ***	Type Three Secretion System	5Â´ CACTAAGCTCGATATATAGAACCC 3’	826 pb	54°C	[20]
		5’ GTCAATGTTGATGTCGTATCTAAG 3’			
***escV***	Type Three Secretion System	5’ GATGACATCATGAATAAACTC 3’	2130 pb	54°C	[20]
		5’ GCCTTCATATCTGGTAGAC 3’			
***traA***	Pilin	5’ AAGTGTTCAGGGTGCTTCTG 3’	385 pb	60°C	[28]
		5’ TATTCTCGTCTCCCGACATC 3’			
***eae***	intimin	5’ CCCGAATTCGGCACAAGCATAAGC 3’	881 pb	52°C	[77]
		5’ CCCGGATCCGTCTCGCCAGTATTCG3’			

### Phenotypic assays

Tests were performed at 37°C to investigate a possible association of curli and cellulose with virulence. Curli production was determined based on colony morphology on CR plates, scored according to the basic morphotypes previously described in *S. typhimurium*[78] rdar (red colony, expresses curli fimbriae and cellulose), pdar (pink colony, expresses cellulose), bdar (brown colony, expresses curli fimbriae) and saw (white colony, no expression of curli fimbriae nor cellulose). CR plates were grown for 24 h. Cellulose production was determined on plates containing 0.025% calcofuor. Fluorescent colonies under a 366 nm UV light source served as an indicator of cellulose production.

The mobility of DAEC strains was determined by the pattern of growth in semi-solid agar.

### Biofilm screening assay and zinc inhibition

In order to screen the biofilm formation by DAEC strains, alone or in association with *C. freundii* 205, 96-well flat-bottom polystyrene plates were used [79]. Briefly, 200 μL per well of DMEM-mannose were inoculated with 5 μL of overnight bacterial culture, and then the plates were incubated overnight at 37°C without shaking. Afterwards, the formed biofilms were stained with CV (crystal violet) for 15 min, washed once with 200 μL of PBS and air-dried for 3 h. The CV adsorbed on the well bottom and the bacterium-bound dye were released by the addition of ethanol (200 μL/well) and the absorbance (OD at 630 nm) was measured. The mean of the absorbances of three independent tests was used as the measure for the formed biofilms. The ability of DAEC strains to form biofilms on abiotic surfaces was assessed by comparison with standard strains that form biofilm (EAEC strain 042 and Cf 205) and a non biofilm forming strain (C600). The *Citrobacter freundii* strain 205 (Cf 205), isolated from a diarrheic child in Brasilia, Brazil [28], was added to controls because it had been used in mixed biofilms assays. Biofilms where divided in two groups according to the optical density comparing to controls. They were considered weak when their OD was within 20% of the Cf205 strain’s; and strong when the OD was greater than that. When the OD was found to be within 20% of the C600 strain’s, it was considered that there was no biofilm formation.

Assays focusing on biofilm inhibition were conducted in the same way using DMEM-mannose containing ZnSO4 at a concentration of 0.25 mM – 12 times lower than the minimum inhibitory concentration (MIC) for zinc [28].

### HeLa cells and infection assays

HeLa cells were cultured in DMEM (DubelcoÂ´s modified Eagle,s medium; Gibco, BRL) with 5% fetal bovine serum and antibiotics (120 μg/mL ampicillin and 100 μg/mL streptomycin) at 4% CO_2_ and 37°C. For qualitative infection assays (adhesion tests), HeLa cells (0.6× 10^5^ cells/mL) were cultured on glass coverslips using 24-well culture plates (600 μL/well) (Costar). Cells were grown to 50%-70% confluence, and the medium was changed to DMEM supplemented with 1% mannose (DMEM-mannose) without FBS. For adhesion assays, HeLa cells were infected with 50 μL of an overnight bacterial culture (OD 0.6 at 600nm) for three hours at 37°C. For mixed infection assays 25 μL of each culture were used. After infection, the coverslips were washed five times with Dulbecco’s PBS (D-PBS). The cells were then fixed with methanol, stained with May-Grünwald and Giemsa stains, and analyzed using light microscopy. DAEC prototype strain C1845 was used as the positive control for the diffuse adhesion phenotype.

### IL-8 secretion

In order to detect IL-8 secretion, after 24h of epithelial cell infection, cell-free culture supernatants were tested in triplicate for this cytokine by enzyme-linked immunosorbent assay using a commercial kit (eBioscience), as recommended by the manufacturer. Samples were considered positive when amounts of IL-8 greater than 10 pg/mL were detected. Non-infected HeLa cells and cells infected with *E. coli* C600 were used as negative controls.

### Scanning electron microscopy (SEM)

For SEM observations, samples were processed following standard protocols. In summary, the samples were fixed overnight at 4°C in Karnovsky’s solution (2.5%. paraformaldehyde, 2% glutaraldehyde in 0.1 M cacodylate buffer, pH 7.4) and then post-fixed with 0.1 M cacodylate buffer (pH 7.4) containing osmium tetroxide (1%) and potassium ferricyanide (0.8%) for 1 h at room temperature. Afterwards, the samples were dehydrated in a graded acetone series (30-100%), dried at critical point using CO2 as the transition fluid, and sputter-coated with gold (2 min).

### Statistical analysis

Results were analyzed using the t test, chi-square, or Fisher’s exact tests, using the most appropriate test for each sample. Results with p-values ≤ 0.05 were considered to be statistically significant.

## Competing interests

The authors declare that they have no competing interests.

## Authors’ contributions

RMA conceived the study and designed the experiments. RMA, ALP and LGG analyzed the data, wrote the manuscript and were responsible for concepts, vision and direction for the study. All authors read and approved the final manuscript.
